# Peter Roy Twentyman

**DOI:** 10.1054/bjoc.2001.2132

**Published:** 2001-11

**Authors:** Tom Connors


*British Journal of Cancer* (2001) **85**(10), 1421
© 2001 Cancer Research Campaign
doi: 10.1054/ bjoc.2001.2132, available online at http://www.idealibrary.com on IDEAL
http://www.bjcancer.com

Obituary

### Peter Roy Twentyman

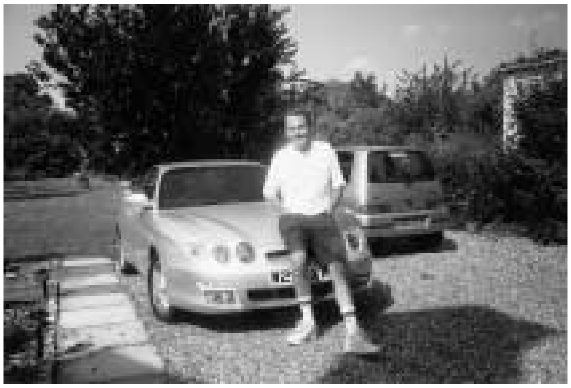

Peter Twentyman died tragically in a light aircraft accident on Wednesday August 15th.

Peter graduated in Physics at Imperial College in 1965 and, after a series of appointments, carried out research leading to a PhD degree in Gordon Steele's department at the Institute of Cancer Research. Peter was appointed as a lecturer in the Radioisotope Unit of the University of Hong Kong from January 1970 to January 1972. Here, Peter was not only active in teaching and research, but was an enthusiastic supporter of the university's cricket club and a wicked hand at darts in the Senior Common Room. His first child, Jessica, was born in Hong Kong. He returned to England to lecture at the Middlesex Hospital Medical School, following which in 1975 he was appointed to the scientific staff of the MRC Clinical Oncology and Radiotherapy Unit, (CORU) at Cambridge. By 1986 he had been promoted to an MRC Special Appointment, the equivalent of a University Professor. During his time at CORU Peter built up an international reputation for his studies on the mechanism of anticancer agents using cells in culture and with particular reference to the development of drug resistance. At the same time he developed a flair for organisation and administration and was invited to join several important committees.

He was a marvellous honorary Secretary of the British Association of Cancer Research from 1985 until 1988 and played a major role in the organisation of the Association's annual and winter meetings. He was instrumental in arranging the successful first collaboration with the European Association for Cancer Research at the 1986 joint winter meeting. Peter was an outgoing character and realised the importance of what nowadays is termed bonding. Thus he was an enthusiastic supporter of Ian Gibson's suggestion of holding the first ever disco at the East Anglia meeting in 1988. I personally found it a great joy if Peter attended a meeting at which I was also a participant. Evenings during meetings where there were no social functions would often be spent in the gloomy bars of student hostels. With the arrival of Peter these evenings would be transformed by his discussions on controversial subjects and would inevitably be concluded by distribution of the BACR songbook.

From 1988 until 1994 he was Editor in Chief of the *British Journal of Cancer*. Again he did a highly professional job, streamlining the journal, introducing new ideas and generally improving its position as one of the leading journals devoted to cancer research and treatment. He was a particular tower of strength in keeping *the Journal* running following the untimely death of the Editor Ged Adams in 1998.

When the Director of a Unit retires, the MRC may decide to close the Unit and this was the decision made on Norman Bleehan's retirement, despite the fact that the Unit was thriving with a number of excellent senior scientists. Although scientists were allowed to continue their research for a period at the Unit this introduced a hiatus in Peter's research and he began to devote more time to administration. In 1996 he was appointed Executive Secretary of the UKCCCR a position he held until he took early retirement in 2000. Peter had planned a full and interesting retirement with his much loved partner Karen and children Jessica, Luke and Daisy. Naturally his early retirement at 58 was honoured by a leaving party that will be remembered by those present for years to come.

Tom Connors

*Received 26 September 2001*
*Accepted 26 September 2001*

